# Genetic Diversity of PCR-Positive, Culture-Negative and Culture-Positive *Mycobacterium ulcerans* Isolated from Buruli Ulcer Patients in Ghana

**DOI:** 10.1371/journal.pone.0088007

**Published:** 2014-02-10

**Authors:** Heather Williamson, Richard Phillips, Stephen Sarfo, Mark Wansbrough-Jones, Pamela Small

**Affiliations:** 1 University of Tennessee, Knoxville, Tennessee, United States of America; 2 Komfo Anokye Teaching Hospital (KATH), Kwame Nkrumah University of Science and Technology (KNUST), Kumasi, Ghana; 3 St. George's, University of London, London, United Kingdom; The University of Hong Kong, China

## Abstract

Culture of *Mycobacterium ulcerans* from Buruli ulcer patients has very low sensitivity. Thus confirmation of *M. ulcerans* infection is primarily based on PCR directed against IS*2404*. In this study we compare the genotypes obtained by variable number of tandem repeat analysis of DNA from IS*2404-PCR* positive cultures with that obtained from IS*2404* positive, culture-negative tissue. A significantly greater genetic heterogeneity was found among culture-negative samples compared with that found in cultured strains but a single genotype is over-represented in both sample sets. This study provides evidence that both the focal location of bacteria in a lesion as well as differences in the ability to culture a particular genotype may underlie the low sensitivity of culture. Though preliminary, data from this work also suggests that mycobacteria previously associated with fish disease (*M. pseudoshottsii*) may be pathogenic for humans.

## Introduction

Buruli ulcer is a necrotizing skin disease prevalent in West Africa and Australia [*1*]. Although culture of *M. ulcerans* has traditionally been the gold standard for diagnosis, growth of the organism can take as long as 8 weeks, facilities for culture are often not available in endemic areas, and sensitivity is low. Even where available, culture has a sensitivity of only 35 to 50% [*2*]. Other methods such as staining for acid-fast bacilli and histology are available (sensitivity is 40% and 63 to 90% respectively), but resources for these methods are lacking in most endemic areas. PCR targeting the insertion sequence IS*2404* has become a rapid and sensitive tool for diagnosis of Buruli ulcer and is now the gold standard for diagnosis. However, culture, microscopy histology, and IS*2404*-PCR cannot be used to identify genetic differences between strains.

The inability to discriminate between different strains of *M. ulcerans* has hampered epidemiological investigations into routes of infection, virulence, and made it impossible to distinguish between relapse and new infection. Molecular epidemiology has benefited from the development of PCR methods based on analysis of restriction fragment length polymorphisms (RFLP), amplified fragment length polymorphisms (AFLP), multi-locus sequence analysis, and PCR of the intragenic regions between insertion sequences IS*2404* and IS*2606* [*3*], [*4*], [*5*]. While these methods could discriminate between strains isolated in widely separated areas such as Australia, China, Japan, Mexico, and Africa, they failed to discriminate between isolates within a specific geographical locale [*3*], [*4*], [*5*].

More recently analysis of variable numbers of tandem repeats (VNTR) within the *M. ulcerans* genome has provided insight into strain variability in African isolates of *M. ulcerans*. PCR targeting two VNTR loci, ST1 and MIRU1, identified three different genotypes among strains isolated from human tissue samples within Ghana [*6*]. The incorporation of two other loci, locus 6 and locus 19, led to more refined sub-grouping and the finding of a fourth genotype [*7*]. VNTR typing has also been used successfully to discriminate between strains of *M. ulcerans* detected in environmental samples collected in Ghana and Benin has also shown strain heterogeneity within aquatic habitats [7], [8], [9].

Genomic data from a number of strains from Ghana has facilitated the development of methods based on the detection of single nucleotide polymorphisms (SNP typing) for studying *M. ulcerans* transmission pathways and phylogenetic relationships [10], [11]. However, these methods have only been used successfully with pure bacterial cultures [10], [11].

A particular advantage of VNTR profiling has been the ability to not only distinguish between *M. ulcerans* genotypes, but also to distinguish *M. ulcerans* from other, recently discovered, mycolactone-producing mycobacteria (MPMs) such as *M. liflandii* and *M. pseudoshottsii* which are pathogenic for aquatic vertebrates (Table 1). These MPMs have been isolated from diseased fish and frogs, but their virulence for humans is not known [*12*], [*13*], [*14*]. Finally, VNTR analysis can be used to identify and type organisms in environmental samples [*7*], [*8*]. VNTR profiling of DNA from aquatic environmental samples collected from Ghana has also led to the discovery that these MPMs can share the same environments with *M. ulcerans* [*7*], [*8*].

**Table 1:** VNTR genotypes of *M. ulcerans*, mycolactone producing *M. marinum and M. pseudoshottsii* (MPM), and *M. liflandii* (MPML).

VNTR Genotype	MIRU1	Locus 6	ST1	Locus 19
*M. ulcerans* Genotypes(Human isolates)				
A	1	1	1	2
B	3	1	1	2
C	3	1	2	2
D	1	1	2	2
*M. marinum/M.pseudoshottsii* Genotype (Fish isolates)				
MPM	1	4	2	2
*M. liflandii* Genotype (Frog isolates)				
MPML	1	2	2	1

VNTR typing was based upon numbers of repeats found at different loci MIRU1, Locus 6, ST1, and Locus 19.

It has been difficult to understand the very low sensitivity of culture from Buruli ulcer patients because *M. ulcerans* is readily grown in the laboratory and the lesions typically contain an extremely high bacterial load. One explanation for the low sensitivity of culture is that the organisms are located in discrete foci within lesions and these foci may be missed through sampling error [15]. A second possibility is that some strains of *M. ulcerans* are not easily cultured.

In order to gain insight into why IS*2404*-positive patient samples containing massive numbers of *M. ulcerans* fail to yield a positive culture, we conducted a study to compare VNTR genotypes of *M. ulcerans* isolated from punch biopsies of Buruli ulcer patients with IS*2404*-positive, culture negative tissues from Buruli ulcer patients.

## Materials and Methods

Two sets of material were used for analysis. The first set of samples contained DNA from punch biopsies of patients with a confirmed diagnosis of Buruli ulcer, but where *M. ulcerans* was not isolated upon culture. Information as to whether a culture of *M. ulcerans* was obtained from the punch biopsy was also included (N = 15). A second set of samples (N = 27) contained DNA isolated from pure cultures of *M. ulcerans* obtained from biopsy material.

From 2006 to 2007, punch biopsy samples were obtained from subjects with Buruli ulcer at the Tepa Government hospital of the Ahafo Ano North District in the Ashanti Region in Ghana. Subjects were provided with study sheets and were recruited only after the study procedures and potential risks associated with participation had been explained and written informed consent obtained. The local ethics committee approved the consent procedure. Ethical approval was obtained from the ethical review committee at the School of Medical Sciences, Kwame Nkrumah University of Science and Technology, Kumasi, Ghana (CHRPE/11/28/06). Samples were processed for diagnostic confirmation by microscopy for acid-fast bacilli, PCR for the IS*2404* insertion sequence of *M. ulcerans* or cultured on Lowenstein-Jensen slopes, as described elsewhere [2].

VNTR analysis targeted four loci: MIRU1, locus 6, ST1, and locus 19. VNTR Primer sequences, PCR thermocycler conditions and controls used are as previously described [*6*], [*7*]. The identity of several positive PCR products from each sample, and any ambiguous bands was confirmed by DNA sequencing. PCR products from positive samples were either cloned into the pCR2.1 Topo vector (Invitrogen), or extracted from the agarose gel using QIAquick Spin (Qiagen) according to the manufacturer's instructions in the instance of a doublet band. In this case both bands were extracted. Sequencing was performed using an ABI 3100 automated genetic analyzer (Applied Biosystems). A matrix of VNTR genotypes representing the number of repeats at each designated locus was used for genotype designation as previously described [7] (Table 1).

The ANOVA and independent t-tests were performed using SPSS 19.0 data analysis software. Significance was defined as p<.05.

## Results

*M. ulcerans* isolates from patient tissues exhibited significantly less genetic variability than those identified from culture-negative tissue samples (p = .012).

VNTR typing was successful for 24/27 (89%) *M. ulcerans* isolates from biopsy tissue from 15 patients. Out of 27 *M. ulcerans* cultures, 23 produced a genotype matching *M. ulcerans* genotype C characteristic of the genome strain, *M. ulcerans* Agy*99* (Table 1, Table 2 and *6,7*). One sample had a VNTR genotype matching *M. ulcerans* genotype A (Table 1 and Table 2). Multiple punch biopsies, taken from individual patients (patients 2,8,11, and 14), yielded identical genotypes (Table 2). A genotype for the 3 samples from patient 15 could not be confirmed because no amplification product was obtained at locus 6 (Table 2). However the single copy at MIRU1 excludes the possibility that the patient was infected with *M. ulcerans* type C. A gel image showing bands from VNTR analysis of representative samples are included as Figure S1.

**Table 2:** VNTR genotypes of *M. ulcerans* isolates from Buruli ulcer punch biopsy tissue.

CULTURE POSITIVE TISSUE SAMPLES					
	VNTR Results				
Patient Number	MIRU 1	Locus 6	ST1	Locus 19	Genotype Designation
1	1	1	1	2	A
2a	3	1	2	2	C
2b	3	1	2	2	C
3	3	1	2	2	C
4	3	1	2	2	C
5	3	1	2	2	C
6	3	1	2	2	C
7	3	1	2	2	C
8a	3	1	2	2	C
8b	3	1	2	2	C
8c	3	1	2	2	C
8d	3	1	2	2	C
9	3	1	2	2	C
10	3	1	2	2	C
11a	3	1	2	2	C
11b	3	1	2	2	C
11c	3	1	2	2	C
11d	3	1	2	2	C
12	3	1	2	2	C
13	3	1	2	2	C
14a	3	1	2	2	C
14b	3	1	2	2	C
14c	3	1	2	2	C
14d	3	1	2	2	C
15a	1	0	2	2	None
15b	1	0	2	2	None
15c	1	0	2	2	None

Multiple samples from a single patient are designated by lower case letters (eg., 2a,2b).

DNA samples from *IS2404*-PCR positive, culture-negative punch biopsies from 15 patients were subjected to VNTR analysis (Table 3). Considerable strain heterogeneity was identified within this sample set. One sample typed as *M. ulcerans* genotype A (Table 1 and Table 3), three samples typed as *M. ulcerans* genotype B (Table 1 and Table 3), eight samples typed as *M. ulcerans* genotype C, and one sample typed as *M. ulcerans* genotype D. Sample 13 matched a VNTR genotype for mycolactone producing mycobacteria (*M. marinum/M. pseudoshottsii*, MPM genotype) associated with fish disease. This genotype has not previously been identified in a human patient. It has recently been proposed that the MPM species should be considered ecovars of *M. ulcerans*. These ecovars have a lower growth temperature requirement than *M. ulcerans* and cannot be isolated under the conditions used to isolate *M. ulcerans* [12], [13], [14], [16]. Sample 15 did not match known genotypes for *M. ulcerans* or MPMs (Table 3). Amplification matched a MPM genotype at three of the four loci, but showed a band higher than control bands at ST1 when viewed on an agarose gel. Sequencing data from this band did not confirm the presence of ST1 DNA.

**Table 3:** VNTR genotypes of *M. ulcerans* culture negative, IS*2404* positive punch biopsy tissue.

CULTURE NEGATIVE TISSUE SAMPLES					
	VNTR Results				
Patient Number	MIRU1	Locus 6	ST1	Locus 19	Genotype Designation
1	3	1	2	2	C
2	3	1	2	2	C
3	3	1	2	2	C
4	3	1	2	2	C
5	3	1	2	2	C
6	3	1	2	2	C
7	3	1	2	2	C
8	3	1	2	2	C
9	3	1	1	2	C
10	3	1	1	2	B
11	3	1	1	2	B
12	1	1	1	2	A
13	1	4	2	2	MPM
14	1	1	2	2	D
15	1	4	HTC	2	NONE

HTC: band was higher than control bands when viewed on an agarose gel.

Strain heterogeneity was much greater among culture-negative tissue samples than in culturable samples (p = .012), and *M. ulcerans genotype* C (p = .037) was cultured more often than any other *M. ulcerans* genotype in the study (p = .037).

## Discussion

This is the first investigation to compare genotypes of *M. ulcerans* from PCR-positive, culture-positive samples with those from PCR-positive, culture negative samples. The low sensitivity of culture for diagnosis [2] has been attributed to the focal distribution of *M. ulcerans* in infected tissue [15]. However, growing evidence on the genetic heterogeneity of *M. ulcerans* raises the possibility that some genotypes are more readily cultured than others. Results in this paper provide evidence for both reasons for culture failure.

*M. ulcerans* genotype C was positively associated with *M. ulcerans* isolation (p = .037). However, this was also the most frequently identified genotype in culture-negative patient tissues. These data provide some evidence that genotype C may be more readily cultured from patient tissue than other genotypes. However, the possibility that genotype C is present in an environment niche associated with a high frequency of human contact could also explain over-representation of genotype C in patient samples.

The fact that VNTR typing showed significantly greater strain heterogeneity among culture-negative biopsy material than among bacterial cultures obtained from patient tissue (p = .012) is particularly interesting. This could be due to the fact that the specific habitats of strains with genotypes A, B, and D are rarely encountered by humans in the environment, or due to the fact that these genotypes do not grow well under laboratory conditions used to culture *M. ulcerans*. The distribution of VNTR genotypes from environmental samples from Ghana is quite different than that obtained from the patient samples analyzed here. VNTR analysis of environmental samples from Ghana shows that 68% of environmental samples from invertebrates, macrophytes, biofilm samples, and water filtrand matched *M. ulcerans* genotype A, 13% matched genotype D, 8% matched *M. ulcerans* genotype B, and 11% matched *M. ulcerans* genotype C (7, and work in progress). In contrast, 74% of the DNA samples genotyped in this study from human tissue samples matched genotype C, while only 4.7%, 7.1% and 2.4% matched genotypes A and B and D respectively. These results suggest more frequent human exposure to the environmental niche associated with genotype C. Although genotypes A, B and D are well represented in the environment, human-environmental contact may not favor exposure to these genotypes.

Laboratory diagnosis of *M. ulcerans* is increasingly based on IS*2404* PCR [17]. Although it was initially reported that *IS2404* was present only in *M. ulcerans*, subsequent work led to the identification of IS*2404* in a closely related group of organisms in the *M. marinum* complex: *M. liflandii*, *M. pseudoshottsii* and a unique clade of mycolactone-producing *M. marinum* that are associated with disease in aquatic amphibians and fish [12], [13], [14], [16]. In addition to containing IS*2404*, these species produce unique forms of the mycolactone toxin. Recent data from whole genome sequencing suggests that all mycolactone-producing mycobacteria should be designated *M. ulcerans* ecovars [18], [19], [20]. However, the pathogenic potential of *M. liflandii, M. pseudoshottsii and* mycolactone producing *M. marinum* for humans is unknown. There is data that mycolactone variants produced by these MPMs have toxicity for human cells [16].

This work provides the first evidence that mycolactone-producing mycobacteria other than *M. ulcerans* could have pathogenic potential for humans. The finding of a VNTR genotype matching that of mycolactone-producing species associated with fish disease (*M. marinum* DL strains and *M. pseudoshottsii*) in IS*2404* positive, culture-negative patient tissue points to the potential virulence of these strains for humans. The fact that a single genotype was isolated from each patient rules out the possibility that the lesion containing a MPM genotype was caused by co-infection with *M. ulcerans*.

A limitation of this work is the small number of patients tested. Our matched sample sets included samples from 15 patients each for a total of 30 patients. Nonetheless, evidence presented here has direct relevance to the poor sensitivity of bacterial culture from *M. ulcerans* patients and suggests that culture sensitivity might be improved by incubating samples at 25 degrees as well as at 32 degrees.

Tim Stinear, lead author on the *M. ulcerans* genome paper suggested that the location of mycolactone genes on a plasmid was evidence that there might be numerous mycolactone producing bacterial groups in the environment, only some with potential for causing human disease [21]. Three DNA samples from *M. ulcerans* isolated from patients with Buruli ulcer had unique VNTR genotypes that did not match known VNTR genotypes. Whether these represent unique mycolactone-producing mycobacteria or simply a failure of amplification could not be determined because of limitations in the amount of sample available.

SNP typing has shown greater strain discrimination among *M. ulcerans* than VNTR typing [10], [11] but has not yet been successfully used on patient samples or environmental samples. Results presented here support the importance of developing finer molecular tools for identification of *M. ulcerans* as well as the importance of including a 25°C incubation temperature for improving isolation of pathogenic mycobacterial species from patient samples.

## Supporting Information

Figure S1 **VNTR of representative tissue samples from patients with a presumptive diagnosis of Buruli ulcer.** (A)VNTR targeting MIRU1. (B)VNTR targeting locus 6. (C)VNTR targeting ST1. (D)VNTR targeting locus 19. All lanes are labeled 1: 1 kb ladder; 2: negative control; 3: Sample showing genotype C (*M. ulcerans* isolated); 4: Sample showing genotype C (*M. ulcerans* not isolated); 5: Sample showing *M. ulcerans* Genotype B (*M. ulcerans* not isolated); 6: Sample showing genotype A (*M. ulcerans* not cultured); 7: Sample showing genotype D (*M. ulcerans* not isolated); 8: Sample showing MPM genotype (*M. ulcerans* not isolated); 9: *M. marinum* DL240490; 10: *M. ulcerans* Agy*99*; 11: *M. ulcerans* 1063; 12: *M. ulcerans* 1059; 13: *M. ulcerans* MK.
(TIFF)
