# Supplementary figures and images for: Genetic Diversity of PCR-Positive, Culture-Negative and Culture-Positive Mycobacterium ulcerans Isolated from Buruli Ulcer Patients in Ghana
Source: PLoS One. 2014 Feb 10;9(2):e88007. doi: 10.1371/journal.pone.0088007 (PMC3919753; doi:10.1371/journal.pone.0088007)

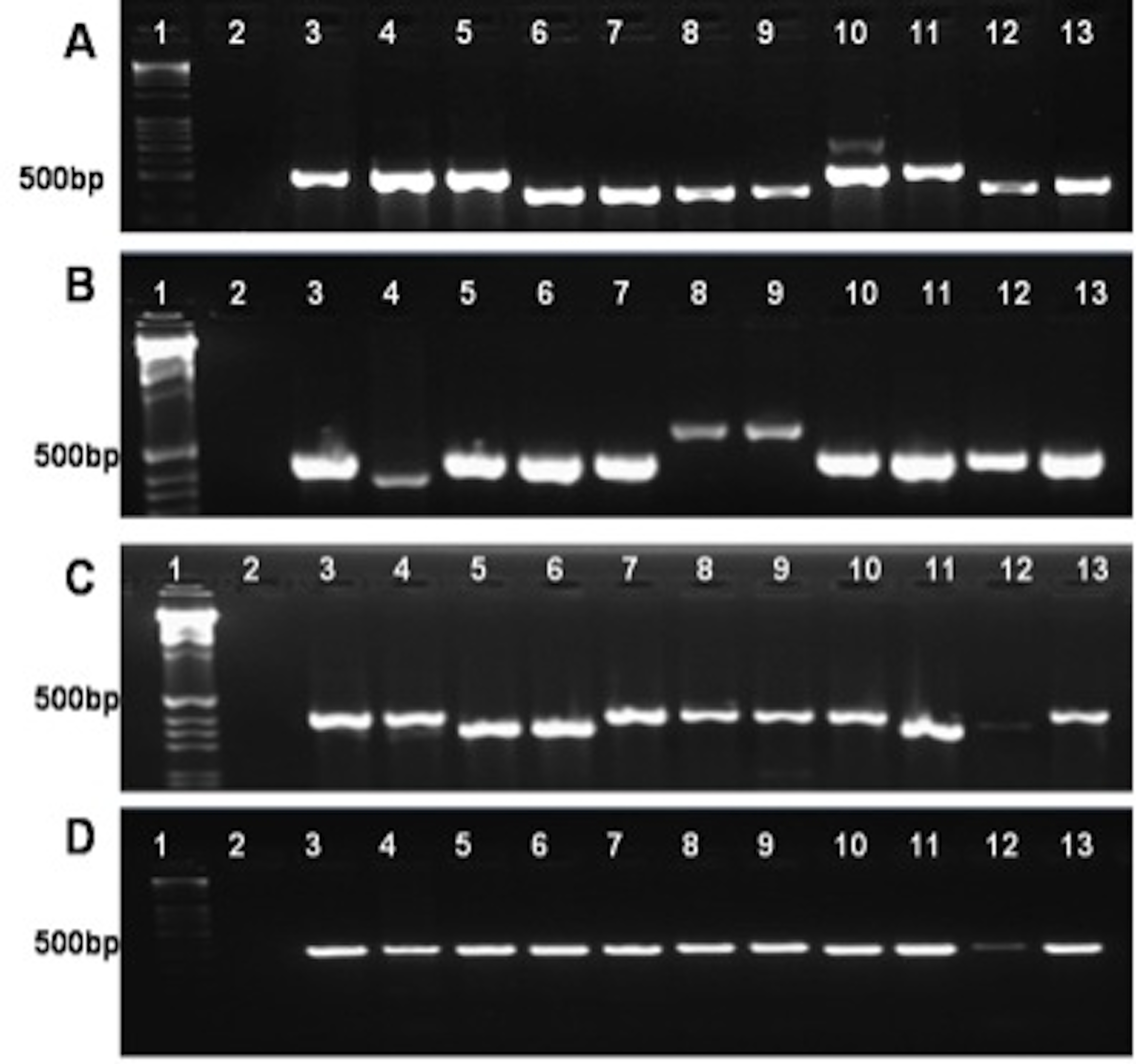

Supplement: Figure S1 — VNTR of representative tissue samples from patients with a presumptive diagnosis of Buruli ulcer. (A)VNTR targeting MIRU1. (B)VNTR targeting locus 6. (C)VNTR targeting ST1. (D)VNTR targeting locus 19. All lanes are labeled 1: 1 kb ladder; 2: negative control; 3: Sample showing genotype C (M. ulcerans isolated); 4: Sample showing genotype C (M. ulcerans not isolated); 5: Sample showing M. ulcerans Genotype B (M. ulcerans not isolated); 6: Sample showing genotype A (M. ulcerans not cultured); 7: Sample showing genotype D (M. ulcerans not isolated); 8: Sample showing MPM genotype (M. ulcerans not isolated); 9: M. marinum DL240490; 10: M. ulcerans Agy99; 11: M. ulcerans 1063; 12: M. ulcerans 1059; 13: M. ulcerans MK. (TIFF) [file pone.0088007.s001.tiff]
